# Epidemiological Profile and Risk Factors for Acquiring HBV and/or HCV in HIV-Infected Population Groups in Nepal

**DOI:** 10.1155/2018/9241679

**Published:** 2018-01-02

**Authors:** Manjula Bhattarai, Jagat Bahadur Baniya, Nirmal Aryal, Bimal Shrestha, Ramanuj Rauniyar, Anurag Adhikari, Pratik Koirala, Pardip Kumar Oli, Ram Deo Pandit, David A. Stein, Birendra Prasad Gupta

**Affiliations:** ^1^Kathmandu Research Institute for Biological Sciences, Lalitpur, Nepal; ^2^National Public Health Laboratory, Teku, Kathmandu, Nepal; ^3^Virology Units, Central Department of Biotechnology, Tribhuvan University, Kirtipur, Kathmandu, Nepal; ^4^Sagarmatha Diagnostic Centre, Nepalgunj, Banke, Nepal; ^5^Ayurveda Campus, TU, Kirtipur, Kathmandu, Nepal; ^6^Department of Biomedical Sciences, Oregon State University, Corvallis, OR, USA

## Abstract

HBV and HCV infections are widespread among the HIV-infected individuals in Nepal. The goals of this study were to investigate the epidemiological profile and risk factors for acquiring HBV and/or HCV coinfection in disadvantaged HIV-positive population groups in Nepal. We conducted a retrospective study on blood samples from HIV-positive patients from the National Public Health Laboratory at Kathmandu to assay for HBsAg, HBeAg, and anti-HCV antibodies, HIV viral load, and CD4+ T cell count. Among 579 subjects, the prevalence of HIV-HBV, HIV-HCV, and HIV-HBV-HCV coinfections was 3.62%, 2.93%, and 0.34%, respectively. Multivariate regression analysis indicated that spouses of HIV-positive migrant labourers were at significant risk for coinfection with HBV infection, and an age of >40 years in HIV-infected individuals was identified as a significant risk factor for HCV coinfection. Overall our study indicates that disadvantaged population groups such as intravenous drug users, migrant workers and their spouses, female sex workers, and men who have sex with HIV-infected men are at a high and persistent risk of acquiring viral hepatitis. We conclude that Nepalese HIV patients should receive HBV and HCV diagnostic screening on a regular basis.

## 1. Introduction

Globally, as of 2015, 240 million people are living with chronic HBV infection [1], 184 million people have chronic HCV infection, and an estimated 36.9 million people are infected with HIV [1–3]. According to the Nepalese National Centre for AIDS and STD Control, as of December 2015, 22,267 people were currently HIV-infected in Nepal. Among them were intravenous drug users (IDUs) (12%), migrant workers (7%), spouses of migrant workers (4%), female sex workers (5%), men who have sex with men (2%), and blood or organ recipients (0.39%) [4]. Data on the infectious burden and clinical severity of HCV and/or HBV coinfection among these key populations of HIV-infected individuals is scant and of uneven quality. Although the dominant transmission mode for HBV, HCV, and HIV infection is different for each virus [5], key population groups such as migrant labourers, female sex workers, and intravenous drug users (IDUs) are common victims of all three viruses [6]. In Nepal, the prevalence of HBV and HCV infection in the general population is 0.47% and 0.64%, respectively [7, 8], dramatically lower than the 3.2% and 4.1% among HIV-infected individuals [9]. IDUs in Nepal have a high-likelihood of being infected with HCV (41.9%), HBV (3.5%), or HIV (13.8%) [10]. Sex workers are also at high risk for HCV, HBV, and/or HIV infection and, according to data from 2003, the number of Nepalese girls and women in Indian brothels exceeds 200,000. HIV-positive female sex workers or sex-trafficked women/girls are more likely to acquire HBV infection than their non-HIV-infected counterparts (9.1% versus 1.4%, resp.) [11]. Further, each year, high unemployment displaces thousands of Nepalese adults to India in search of livelihood [12] and upon returning home, many carry sexually transmitted infections [13]. As a result, HBV and HIV prevalence among such migrant workers has been reported as high as 11% and 8%, respectively [14, 15].

Many individuals in disadvantaged population groups lack awareness of and are poorly educated about preventive measures for infection-avoidance. Unfortunately, the postinfection clinical and health status for many of these individuals is unknown. Contemporary data regarding infection and health status inside these populations of HIV-infected individuals is poor. The collection and analysis of an increased amount of scientifically sound data are imperative to inform the design of public-health policies and to help establish more effective outreach programs. This current study aims to investigate the epidemiological features of HBV and/or HCV coinfection among HIV-infected populations and thereby help identify risk factors for coinfection.

## 2. Materials and Methods

### 2.1. Study Population

A retrospective study was conducted using blood samples collected from HIV-positive individuals at the Nepalese National Public Health Laboratory (NPHL), Nepal, during January–December 2015. With consent from NPHL officials, 682 samples were retrieved blindly from the blood sample archive, and 579 were deemed fit for our study. Relevant demographic information and laboratory values were retrieved from the clinical record files of each individual patient. Exclusion criteria included age below 18 years, plasma sample volume <50 *μ*l, and lack of updated clinical history including CD4+ T cell count, viral load, BMI, and ART regime used. All individuals were from seven (Baglung, Banke, Chitwan, Kailali, Kaski, Kathmandu, and Tanahun) of the 75 current districts of Nepal. As controls, we used blood samples and demographic data of 581 healthy volunteers aged ≥18 years. These blood samples were collected between February and March 2014. This control group consisted of blood samples from 401 male and 180 females. Among the screened samples, 37 were from residents of the Kathmandu valley and 544 were from outside the valley.

### 2.2. Blood Work-Up

At the time of patient visit, 5 ml venous blood was drawn by a certified phlebotomist and collected in EDTA tubes (BD, USA). The collected whole blood (1/3rd) underwent CD4+ T cell count, and the remainder was centrifuged at 3000 rpm for 5 min at RT. After centrifugation, blood plasma (2/3rd) was used for routine biochemical and HIV viral load assays, while the remaining plasma (1/3rd) was transferred to 1.8 ml cryo-vial (Abdos, India) and stored at −20°C until further analysis.

### 2.3. CD4+ T Cell Count

Twenty microliters of Tritest CD3/CD4/CD45 reagent (BD, USA) along with 50 *μ*L of well-mixed anticoagulated whole blood was pipetted into the bottom of a Trucount tube (BD, USA) labelled with sample identification number. After gentle vortexing, the tube was incubated for 15 min in the dark at RT. After incubation, 450 *μ*L 1x FACS lysing solution (BD, USA) was added to the tube. The tube was again gently vortexed and incubated for 15 min in the dark at RT before analysis on a FACSCalibur (BD, USA).

### 2.4. HIV Viral Load Assay

HIV RNA was isolated from 140 *μ*l of plasma using Nucleospin viral RNA isolation kit (MACHEREY-NAGEL, Germany) according to the manufacturer's instruction. For viral RNA amplification and cDNA preparation, Artus HI Virus-1 QS-RGQ Kit (QIAGEN, Germany) was used with primers and internal controls supplied by the manufacturer in a complete master mix in the kit.

### 2.5. ELISA

Plasma samples stored at −20°C were thawed to room temperature (25°C) and used for HBsAg, HBeAg, and anti-HCV antibody detection by ELISA (Wantai Co., China). All ELISA was performed under sterile condition according to manufacturer's instructions. Positive and negative controls were supplied in the kit and the cut-off values for the respective tests were defined according to the manufacturer's instructions.

### 2.6. Statistical Analysis

All data was analysed using SPSS software version 23.0. Frequencies were calculated for categorical variables and mean ± SD were calculated for quantitative variables. *P* values of <0.05 were considered significant, unless otherwise noted. Odds ratios with 95% confidence intervals were calculated. Categorical variables were compared using chi-squared test, while paired *t*-test was used for comparing continuous variables. Spearman's correlation coefficient was used to see the degree of correlation between continuous variables. Univariate and multivariate analysis using stepwise regression analysis was done on the described variables.

## 3. Results

### 3.1. Epidemiological Features of HIV-Infected Patients Coinfected with HBV or HCV

Of the 579 HIV-positive patients enrolled in this study, 72.19% were men and 0.34% transgender, with a male/female ratio of 2.6 : 1. The age-range of the study subjects was 18–65 years with a mean of 39.13. The prevalence of HIV-HBV, HIV-HCV, and HIV-HBV-HCV coinfections was found to be 3.62%, 2.93%, and 0.34%, respectively. HIV-positive males were coinfected with either HBV or HCV at a higher rate (2.2%) than HIV-positive females (1.3%). Adults 21–59 years of age were observed to be at increased risk of HBV and/or HCV coinfection compared to those in younger (18–20 years) and older (60–65 years) age groups (*P* < 0.05). Marital status of the individual had a positive correlation with HCV coinfection (*P* < 0.05); however, the same did not hold for HBV coinfection. In addition, the IDU population was observed to be HCV-infected at a far higher frequency compared to other key population groups (Table 1) (*P* < 0.05). All the 579 HIV-positives enrolled were on antiretroviral therapy (ART) at the time of study and none had received direct-acting anti-HCV medications (e.g., Sofosbuvir) or peginterferon for at least 4 months prior to blood sample collection.

### 3.2. Risk Factors for Coinfection with HBC or HCV in HIV-Positive Individuals

The subject's age, body mass index, sex, geographical location, current ART regime, key population category, HIV viral load, education level, marital status, ART duration, and CD4+ T cell count were included in a univariate logistic regression model, where HIV/HCV and HIV/HBV coinfection were dependent variables. The univariate analysis showed that being a spouse of a migrant labourer (OR: 4.33, 95% CI: 1.03–21.65), female sex worker (OR: 4.96 95% CI: 0.62–31.61), intravenous drug user (OR: 1.45, 95% CI: 0.26–7.96), male sex worker (OR: 2.01 95% CI: 0.49–10.13), or having an HCV positive status (OR: 3.81, 95% CI: 0.57–14.84) positively correlated with HIV/HBV coinfection. Additionally, an age > 40 years (OR: 1.04, 95% CI: 1.00–1.09) or male gender (OR: 1.24, 95% CI: 0.43–4.46) positively correlated with HIV/HCV coinfection (*P* < 0.05) (Table 2).

Risk factors observed by the univariate model were then analysed in a multivariate logistic regression model using stepwise regression analysis. Multivariate analysis showed a higher prevalence of HIV/HBV coinfection in certain key population groups, such as female sex worker (OR: 5.25, 95% CI: 0.82–33.60) and spouses of migrant labourers (OR: 4.01, 95% CI: 0.92–17.40), than in other at-risk population groups (Table 3). Although a higher prevalence of HIV/HCV coinfection was observed in individuals of >40 year of age (OR: 1.04, 95% CI: 1.00–1.09), HIV-positives having CD4+ T cell counts of >200 cells/*μ*L were associated with lowered risk of coinfection with HCV (OR: 0.19, 95% CI: 0.06–0.52) (Table 3), suggesting that age and CD4+ T cell count may affect the risk for HIV/HCV coinfection.

## 4. Discussion

In South Asia, the prevalence of HBV and/or HCV seropositivity among HIV-positives varies distinctively by location. In North India, HBV seropositive cases are reported as high as 6.2% and HCV seropositive cases as low as 1.56% among HIV-infected individuals [16]. Our study reveals a higher prevalence of HCV/HIV-positive cases compared to North India, perhaps due to the higher prevalence of IDUs in the region of Nepal where our study was conducted [10]. However, in another study among IDUs of West India, the prevalence for HCV or HBV coinfection with HIV was as high as 92% and 100%, respectively [17]. This discrepancy underscores the observation that HIV-positive IDUs are at a high risk of acquiring HCV infection compared to other HIV-positive risk groups. This conclusion is further supported by our study in which 88.23% of the total HCV positive cases were IDUs (Table 1). A similar study from Thailand reported that being HIV-positive did not have a significant effect on acquiring HCV infection but that a low CD4+ T cell count was associated with HBV infection [18]. In our study, a higher CD4+ T cell count (>200 cells/*μ*L) was associated with a decreased risk of HCV or HBV coinfection among HIV-positives (Tables 2 and 3). A contributing factor to our observation that HCV-negatives had higher CD4+ T cell counts than HCV-positives could be an increased differentiation of CD4^+^ to Th17 effector cells in HCV-infected hepatocytes [19, 20].

Among HIV-positives worldwide, key disadvantaged populations have a more frequent exposure to HBV and HCV infection due to engagement in high-risk behaviours, weak family and social support systems, and inadequate access to healthcare services [3]. Additionally, negative social-stigma and association with infection-positive status undermine efforts for initial and follow-up diagnosis, thus impeding preventive measures against the spread of all three viral diseases. Female sex workers, who are vulnerable to sexually transmitted diseases, have been reported to have HCV infection rates of 8.1% in Iran to 8.8% in Brazil [21, 22], suggesting that, regardless of culture, geography, and GDP, HCV infection has a consistent prevalence among that cohort. Of note, in our study, being the spouse of a migrant worker was even more of a risk factor for HBV and/or HCV infection than being a female sex worker. Our study also revealed that women who have not completed primary schooling and whose husbands are working abroad are at an increased risk of infection and hence should be advised to receive regular screening for viral hepatitis and/or HIV.

A study carried out in Nepal during 2010-2011 reported that the prevalence of HBV and HCV confections among the HIV-positive population was 4.4% and 19%, respectively [23]. Those reported rates are both significantly higher than those of our current study. One possible reason for this discrepancy could be that over the past few years improved diagnostic measures have been implemented by the Nepalese Health Ministry to manage HIV-associated coinfections. Alternatively, it may also be due to design-limitations in our study, in that our total sample size is relatively small and is representative of a geographic subsection of Nepal containing a lower overall prevalence of HCV/HBV than the entire country overall. Additionally, the migrant-worker husbands of the female spouses surveyed in our study were not included in our analysis, due to their unavailability, and it is likely that they represent a cohort having high coinfection rates. Nevertheless, this study considerably extends our understanding of the risk factors for coinfection in Nepal. Hopefully the data and analysis presented here can contribute to crafting future policies designed to impede the transmission of HBV and HCV in general and to HIV patients in particular. Our surprising observation that increased CD4 count correlated as a risk factor for HCV coinfection suggests that additional research is necessary to address the issue of HCV-progression in HIV-positive individuals who are receiving ART. Interestingly, a higher CD4 count was not associated with HBV coinfection.

Our study shows that coinfection with HBV and/or HCV is a serious health concern for HIV-infected members of disadvantaged population groups in Nepal. Further, our study suggests that spouses of migrant workers and female sex workers have increased risk of acquiring HBV and/or HCV. We highly recommend improved measures to ensure routine screening for HBV and HCV infection among HIV patients and their family members in Nepal. Such a diagnostic strategy could serve to decrease overall disease prevalence and promote better health among both at-risk groups and the general population.

## Figures and Tables

**Table 1:** Epidemiological characteristics of HBV and HCV infections in 579 HIV-infected patients in Nepal.

Characteristic	Anti-HBsAg^+^ (percentage, %) (*N* = 21)	Anti-HCV IgM^+^ (percentage, %) (*N* = 17)
*Age (years)*		
<20	5 (0.86)	1 (0.17)
21–40	7 (1.20)	6 (1.03)
41–59	8 (1.38)	8 (1.38)
>60	1 (0.17)	2 (0.34)
Total	21	17
*χ* ^2^	4.422	7.853
*P*	0.219	0.049^*∗*^
*Sex*		
Male	13 (2.24)	13 (2.24)
Female	8 (1.38)	4 (0.69)
Transgender	—	—
*χ* ^2^	1.292	0.203
*P*	0.524	0.903
*CD4 count (cells/μL)*	403.19 ± 328.53	272.04 ± 181.82
Marital status		
Married	13 (2.24)	14 (2.41)
Unmarried	8 (1.38)	2 (0.34)
Divorced	—	1 (0.17)
Widowed	—	—
*χ* ^2^	5.583	10.304
*P*	0.134	0.016^*∗*^
*Education*		
Postsecondary school	3 (0.51)	1 (0.17)
Secondary school	9 (1.55)	8 (1.38)
Primary school	4 (0.69)	3 (0.51)
Illiterate	5 (0.86)	5 (0.86)
*χ* ^2^	2.965	5.468
*P*	0.397	0.141
*Key population*		
Labour migrants	3 (0.51)	—
Blood transfusion	1 (0.17)	—
Female sex worker	2 (0.34)	—
Intravenous drug user	3 (0.51)	15 (2.59)
Male sex worker	5 (0.86)	2 (0.34)
Spouse of labour migrants	5 (0.86)	—
Transvertical	2 (0.34)	—
*χ* ^2^	20.83	51.564
*P*	0.022	0.000^*∗*^

__^*∗*^ **P** < 0.05 is considered as significant.

**Table 2:** Univariate analysis of factors affecting HBV and HCV infection in 579 HIV-infected subjects in Nepal.

Parameters	HIV HBV coinfection		HIV HCV coinfection	
	OR	*P* value	OR	*P* value
*Age (years)*				
≤40	1		1	
>40	0.9975 (0.964–1.033)	0.888	1.04 (1.00–1.09)	0.0311^*∗*^
*BMI*	0.98 (0.86–1.12)	0.864	0.90 (0.77–1.05)	0.209
*Gender*				
F	1		1	
M	0.60 (0.25–1.56)	0.275	1.24 (0.43–4.46)	0.707
Transgender		0.99	—	0.991
*Geography*				
Baglung	1		1	
Banke	0.36 (0.04–0.73)	0.377	—	
Chitwan	0.07 (0.002–2.01)	0.0778	—	
Kailali	0.12 (0.004–3.48)	0.1633	—	
Kaski	0.21 (0.024–4.51)	0.1973	—	
Kathmandu	0.17 (0.020–3.76)	0.1496	—	
Tanahun	0.45 (0.03–1.09)	0.5506	—	
*Regime*				
1st	1		1	
2nd	4.09 (0.61–16.07)	0.075	2.27 (0.12–12.37)	0.439
*Key population*				
Labour migrants	1		1	1
Blood transfusion	—		—	1
Female sex worker	4.96 (0.62–31.61)	0.0883	—	1
Intravenous drug user	1.45 (0.26–7.96)	0.6519	—	0.99
Male sex worker	2.01 (0.49–10.13)	0.33	—	0.99
Spouse of labour migrants	4.33 (1.03–21.64)	0.0489^*∗*^	—	1
Transvertical	4.20 (0.53–26.55)	0.1253	—	1
*Viral load*	1.01 (0.94–1.06)		1.01 (0.92–1.06)	0.707
*HCV status*				
Negative	1			
Positive	3.81 (0.57–14.84)	0.0896		
*HBV status*				
Negative			1	
Positive			3.81 (0.57–14.84)	0.0896
*Education*				
Illiterate	1		1	
Postsecondary	0.57 (0.11–2.40)	0.457	0.18 (0.009–1.19)	0.131
Primary	0.63 (0.15–2.46)	0.511	0.47 (0.09–1.97)	0.316
Secondary	1.44 (0.48–4.81)	0.517	1.27 (0.41–4.32)	0.674
*Marital status*				
Divorced	1		1	
Married			0.06 (0.005–1.38)	0.0268^*∗*^
Unmarried			0.03 (0.002–0.09)	0.0808
Widowed	—	1	—	0.9894
*ART duration (≥3 years)*	0.79 (0.62–0.97)	0.0399	0.99 (0.81–1.19)	0.946
*CD4 count*				
<200	1		1	
>200	0.52 (0.19–1.62)	0.214	0.17 (0.06–0.47)	0.00047^*∗*^

^*∗*^ **P** < 0.05 is considered as significant.

**Table 3:** Multivariate analysis of factors affecting HBV and HCV infection in 579 HIV-infected subjects in Nepal.

HIV HBV				HIV HCV			
Parameters	OR	95% CI	*P* value		OR	95% CI	*P* value
Blood transfusion	0.24	0.12–0.44	—	*Age*	1.04	1.00–1.09	0.0454^*∗*^
Female sex worker	5.25	0.82–33.60	0.0797	Marital status (married)	0.18	0.01–2.82	0.2263
General population	0.0000	—		Marital status (unmarried)	0.23	0.01–5.14	0.3560
Housewife	0.0000	—		Marital status (widowed)	—	—	0.9905
Intravenous drug user	1.45	0.28–7.35	0.6512	CD4 count > 200 cells/mm^3^	0.19	0.06–0.52	0.0015^*∗*^
Male sex worker	2.08	0.48–8.89	0.3230				
General spouse	0.0000	—					
Spouse of intravenous drug user	0.0000	—	0.9953				
Spouse of migrant	4.01	0.92–17.40	0.0233^*∗*^				

^*∗*^ **P** < 0.05 is considered as significant.

## Abbreviations

HBV: Hepatitis B virus
HCV: Hepatitis C virus
AIDS: Acquired immunodeficiency syndrome
STD: Sexually transmitted diseases
FSW: Female sex worker
IDU: Intravenous drug user
MSW: Male sex worker
HIV: Human immunodeficiency virus
IDU: Intravenous drug user
ART: Antiretroviral therapy
CD4: Cluster of differentiation
BMI: Body mass index
EDTA: Ethylene diamine tetra acetic acid
RT: Room temperature
FACS: Fluorescence activated cell sorter
cDNA: Complementary deoxyribose nucleic acid
Th 17: T helper 17 cell.

## Additional Points

*Availability of Data*. The datasets used and/or analysed during the current study are available from the corresponding author upon reasonable request.
